# Targeted Therapy and Immunotherapy Response Assessment with F-18 Fluorothymidine Positron-Emission Tomography/Magnetic Resonance Imaging in Melanoma Brain Metastasis: A Pilot Study

**DOI:** 10.3389/fonc.2018.00018

**Published:** 2018-02-22

**Authors:** Nghi C. Nguyen, Melissa K. Yee, Abuzar M. Tuchayi, John M. Kirkwood, Hussein Tawbi, James M. Mountz

**Affiliations:** ^1^Department of Radiology, University of Pittsburgh Medical Center, Pittsburgh, PA, United States; ^2^Department of Medicine, Division of Hematology/Oncology, University of Pittsburgh Medical Center, Pittsburgh, PA, United States; ^3^Department of Melanoma Medical Oncology, Division of Cancer Medicine, The University of Texas MD Anderson Cancer Center, Houston, TX, United States

**Keywords:** F-18 fluorothymidine–, PET/MRI, melanoma brain metastasis, targeted antitumor therapy, immunotherapy, early response evaluation

## Abstract

**Introduction:**

This pilot study aimed at exploring the utility of the proliferation tracer F-18 fluorothymidine (FLT) and positron-emission tomography (PET)/magnetic resonance imaging (MRI) (FLT–PET/MRI) for early treatment monitoring in patients with melanoma brain metastasis (MBM) who undergo targeted therapy or immunotherapy.

**Material and Methods:**

Patients with newly diagnosed MBM underwent baseline and follow-up FLT–PET/MRI scans at 3–4 weeks of targeted therapy or immunotherapy. Up to six measurable brain lesions ≥1.0 cm per subject, as identified on T1-weighted post-gadolinium images, were included for quantitative analyses. The maximum SUV of each lesion was divided by the mean SUV of the pons to obtain the SUV ratio (SUVR).

**Results:**

Five enrolled subjects underwent the baseline FLT–PET/MRI study in which the MBM showed a median size of 1.7 cm (range 1.0–2.9) and increased metabolic activity with SUVR of 9.9 (range 3.2–18.4). However, only two subjects (cases #1 and #2) returned for a follow-up scan. At baseline, a total of 22 lesions were analyzed in all five subjects, which showed a median size of 1.7 cm (range 1.0–2.9) and median SUVR of 9.9 (range 3.2–18.4). At follow-up, case #1 was a 55-year-old man who received targeted BRAF inhibitor and MEK inhibitor therapy with dabrafenib and trametinib. Fused PET/MRI data of six measured lesions demonstrated a significant reduction in MBM proliferative activity (median −68%; range −38 to −77%) and size (median −23%; range −4 to −55%) at three weeks of therapy. Nevertheless, the subject eventually progressed and died 13 months after therapy initiation. Case #2 was a 36-year-old man who received immunotherapy with nivolumab and ipilimumab. The five measured MBM lesions showed a mixed response at both proliferative and morphologic imaging at 1-month follow-up. Some lesions demonstrated interval decrease while others interval increase in proliferative activity with a median −44% (range −77 to +68%). On MRI, the size change was +7% (range −64 to +50%). The therapy was switched to dabrafenib and trametinib, which led to a partial response. The patient is still alive 16 months following therapy initiation.

**Conclusion:**

The five cases presented show the potential benefit of hybrid FLT–PET/MRI for the diagnosis of MBM and treatment monitoring of targeted therapy and immunotherapy. However, further studies are required to assess their complementary role in distinguishing true progression from pseudoprogression.

## Introduction

Cutaneous malignant melanoma is the most aggressive form of all skin cancers. It is estimated that there will be doubling of the incidence of melanoma every 10–20 years. Approximately 132,000 people are diagnosed with melanoma each year worldwide, and it causes about 37,000 deaths annually (1). Melanoma has a particular predilection toward distant metastases by simultaneous lymphatic and hematogenous spread. Approximately 40–50% of stage IV melanoma patients eventually develop clinical manifestations of melanoma brain metastasis (MBM).

Melanoma is the third most common cause of metastatic brain metastasis development. Although outcomes differ for patients with MBM, overall prognosis remains poor with 5-year overall survival of less than 10% and a median survival of less than 1 year (2). The prognosis of melanoma brain metastases is poor despite advances in systemic therapies (3–7). There is a sense of urgency to establish novel methods for predicting early response to therapy in MBM because, despite early diagnosis and aggressive local therapy, metastatic brain lesions remain the cause of death in the majority of these patients (95%). As a result, patients with MBM are often excluded from clinical trials.

While new targeted therapies and immunotherapies are now available for MBM, the efficacy of these agents has yet to be established (8). The overall survival of MBM patients reflects the effects of therapy on both intracranial and extracranial disease at the time of presentation; however, measuring extracranial disease routinely may not be entirely representative of intracranial disease control. Contrast-enhanced magnetic resonance imaging (MRI) is a well-established imaging modality in the clinical and research setting. It has numerous clinical applications and is the neuroimaging gold standard for the assessment of CNS neoplasms owing to its superb anatomical detail (9). However, the post-therapy viability of intracranial tumors has been difficult to reliably assess as brain lesions may appear larger in the setting of radiation necrosis and pseudoprogression which may be encountered in as much as 15% of cases (10–15).

Although F-18 fluorodeoxyglucose (FDG) is the most commonly used positron-emission tomography (PET) radiotracer in oncology, its high physiologic brain uptake limits the delineation of a tumor from normal brain metabolic activity; thus, FDG–PET is considered suboptimal for tumor response evaluation (16). There is an increasing clinical and research interest in applying other PET agents to avoid the shortcomings of FDG–PET. F-18 fluorothymidine (FLT) is an analog to the nucleoside thymidine and was developed as a PET agent to assess cellular proliferation by tracing the thymidine salvage pathway (17). A benefit of FLT over FDG is the negligible uptake in the normal cerebral parenchyma, leading to better lesion-to-background ratio and as a result better detection and characterization of brain tumors (18, 19). The potential of FLT–PET in predicting treatment response in malignant gliomas as well as advanced extracranial melanoma treated with the anti-CTLA4 antibody tremelimumab has been demonstrated (19–23). The aim of this pilot study is to explore the utility of hybrid FLT–PET/MRI for early treatment monitoring in patients with MBM who are undergoing targeted therapy or immunotherapy. We hypothesized that the integration of more advanced biomarkers, such as FLT–PET and contrast-enhanced MRI, would provide a complementary evaluation of both cell proliferation and morphology in patients with MBM.

## Materials and Methods

### Study Population

Adult patients with newly diagnosed MBM selected for targeted systemic therapy (dabrafenib, trametinib) or immunotherapy (ipilimumab, nivolumab) were eligible for enrollment in this pilot study. Enrolled subjects would undergo baseline and follow-up FLT–PET/MRI scans performed at least 2 weeks after therapy initiation on a hybrid PET/MRI scanner (Biograph mMR, Siemens). Subjects were excluded if they had an allergy to gadolinium, estimated creatinine clearance <30 ml/min/1.73 m^2^, were severely claustrophobic, or had MRI-incompatible implanted pacemaker or other metallic devices. This study was carried out in accordance with the recommendations of the University of Pittsburgh Institutional Review Board, with written informed consent from all subjects. All subjects provided a written informed consent for their participation in this study and for their personal information to be used for research and publication. Written informed consent was obtained in accordance with the Declaration of Helsinki.

### PET/MRI Scanning

Anatomical pre- and post-gadolinium (Gd) MR images included T1-weighted, T2-weighted, and fluid-attenuated inversion recovery sequences. Each PET acquisition commenced with IV injection of approximately 5 mCi FLT and lasted for a total of 78 min. Due to an acquisition system malfunction, the PET sinograms and list mode data from one of the scans were not available for offline processing. Thus, the results reported were obtained using the default reconstruction performed automatically at the time of data acquisition. For each acquisition, the full 78-min scan was reconstructed into a single frame image using ordinary-Poisson-ordered subset expectation maximization with three iterations and 27 subsets. MRI-based attenuation correction was performed based on either the Dixon method or ultrashort echo time sequences (UTE), implemented consistently at baseline and follow-up scan (24, 25). Additional corrections included corrections for normalization, scatter, random, and dead-time losses. The final PET images had a spatial resolution of 5–6 mm.

### Image Analysis

Positron-emission tomography (PET)/MR images were reviewed and analyzed on a MIM workstation, version 6.1 (MIM Software Inc., Cleveland, OH, USA). Two experienced nuclear radiologists interpreted the PET/MR images qualitatively, and the interpretation of treatment response was determined based on consensus. Subsequently, the quantitative analysis was performed by one of the two nuclear radiologists. Up to six brain lesions ≥1.0 cm in largest diameter as identified on T1-weighted post-Gd images were included for quantitative analyses. The analyses included the largest lesion diameter and maximum standardized uptake value (SUV) at baseline and follow-up PET/MRI scans.

The maximum SUV of each lesion was divided by the mean SUV of the pons obtained using a 1.5 cm ROI placed in the central aspect of the pons unless mentioned otherwise. The intent of producing this SUV ratio (SUVR) was to normalize for potential differences in vascular FLT content between PET scans to partially compensate for the fact that the PET images were averaged over the full acquisition and include contributions from the vascular phase of the tracer. The pons was not involved by tumor in four of five subjects. Case #4, who underwent the baseline scan only, showed a 0.3 cm contrast-enhancing lesion in the left side of the pons which, however, was not FLT avid; the ROI was placed to the right side of the pons to obtain the SUV in this case. The % change in lesion metabolic activity (Δ*SUVR*%) as measured by SUVR was calculated as follows:

ΔSUVR%=[SUVR(follow-up)−SUVR(baseline)]/SUVR(baseline)×100.

### Statistical Analysis

Descriptive statistics (median, minimum–maximum) were used to summarize the lesion size and SUVR findings. No specific statistical test was performed due to the small sample size.

## Results

Five enrolled subjects underwent the baseline FLT–PET/MRI study; however, only two subjects (cases #1 and #2) returned for follow-up scan.

### Subjects with Baseline and Follow-up Scans

#### Case #1

A 55-year-old man who presented with a headache and was found to have multiple brain lesions on MRI suspicious for metastasis. Diagnostic CT of the torso showed an indeterminate 6 mm pulmonary nodule in the right lower lobe, and a 1.8-cm left axillary lymph node. An excisional biopsy of the left axillary node showed metastatic melanoma with extensive large cell involvement, S100 positive, Melan-A positive, and HMB-45 positive. The patient was started on corticosteroids and levetiracetam. Mutation analysis revealed a BRAF V600E mutation. He was subsequently initiated on combination targeted therapy with the BRAF inhibitor dabrafenib (150 mg BID orally) and the MEK inhibitor trametinib (2 mg QD orally).

**Figure 1:**
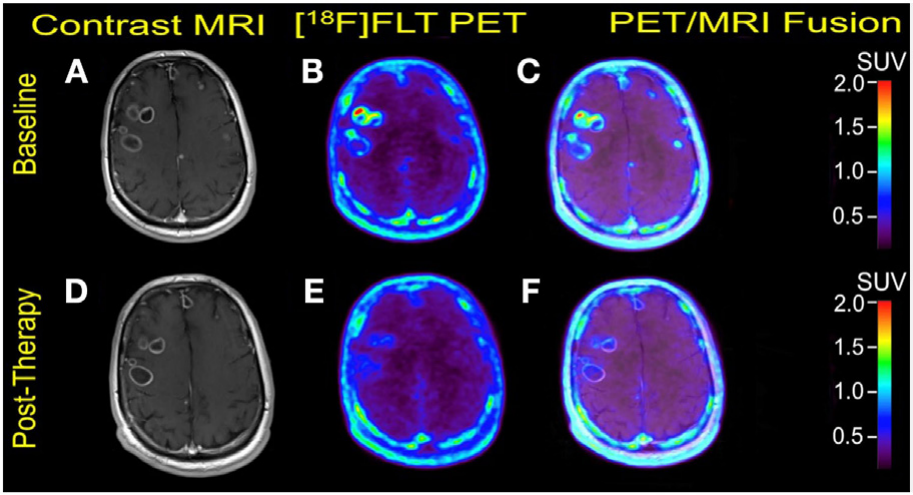
Case #1. Baseline magnetic resonance imaging (MRI) **(A)**, fluorothymidine (FLT)–positron-emission tomography (PET) **(B)**, and fused PET/MRI **(C)**; follow-up MRI **(D)**, FLT–PET **(E)**, and fused PET/MRI **(F)** at 3 weeks. Bilateral supratentorial brain lesions of greater than 1 cm demonstrate variable mild to intense FLT avidity. They showed a greater proliferative reduction compared with the size reduction at 1-month follow-up. Gadolinium-enhanced T1-weighted spin echo with TR 708 ms, TE 8.4 ms, and 90° flip angle, 5 mm slice thickness.

Multiple cerebral lesions demonstrated variable mild to intense FLT uptake at baseline, followed by a marked reduction in FLT uptake at 3 weeks of therapy; however, most lesions still showed mild FLT uptake higher than that of the pons background suggestive of partial treatment response or possibly proliferation of inflammatory cells at the sites of tumor response (Table 1). The six measured lesions showed −68% (range −38 to −77%) median reduction in proliferative activity at 1-month follow-up; there was no new FLT avid cerebral lesion. The median SUVR was 8.3 (range 6.4–11.9) at baseline and 2.7 (range 2.4–4.4) at follow-up scan. Compared to PET findings, the size reduction on MRI was lower with a median of −23% (range −4 to −55%), and there was no evidence of new metastasis (Figure 1). A standard-of-care clinical brain MRI scan obtained 8 weeks later showed a further reduction in the size of MBM.

**Table 1:** Case #1, size (cm) and SUV ratio (SUVR) of six representative melanoma brain metastases (MBM).

Size (baseline)	Size (follow-up)	Change in %	SUVR (baseline)	SUVR (follow-up)	Change in %
1.8	1.1	−39	9.6	2.4	−75
1.1	0.6	−45	9.6	2.8	−71
1.4	1.2	−14	6.4	2.7	−59
2.3	2.2	−4	6.4	2.5	−61
1.2	0.9	−25	7.1	4.4	−38
1.1	0.5	−55	11.9	2.7	−77

Unfortunately, the patient developed seizure 6 months following the therapy and was found to have progressive brain metastasis at brain MRI, which required a change in treatment. Additional treatment with robotic radiosurgery (Cyberknife™) with 21 Gy in a single fraction and therapy with one cycle of ipilimumab (300 mg IV) and three cycles of pembrolizumab (183 mg IV) during 6 weeks were attempted; he, however, died 13 months after the targeted therapy’s initiation.

#### Case #2

A 36-year-old man was initially diagnosed a melanoma on his scalp with wide local excision in September 2003. A sentinel lymph node biopsy and all seven excised nodes were negative for metastasis, stage T4N0Mx. In August 2015, the patient noted some cognitive difficulties and developed a seizure and was found to have multiple brain lesions on MRI and FDG-PET/CT scans. Surgical resection of the large, hemorrhagic lesion showed metastatic melanoma with BRAF V600E mutation. He was then enrolled in a phase II trial to investigate the use of a combination of a PD1 inhibitor (nivolumab) and an anti-CTLA4 antibody (ipilimumab).

**Figure 2:**
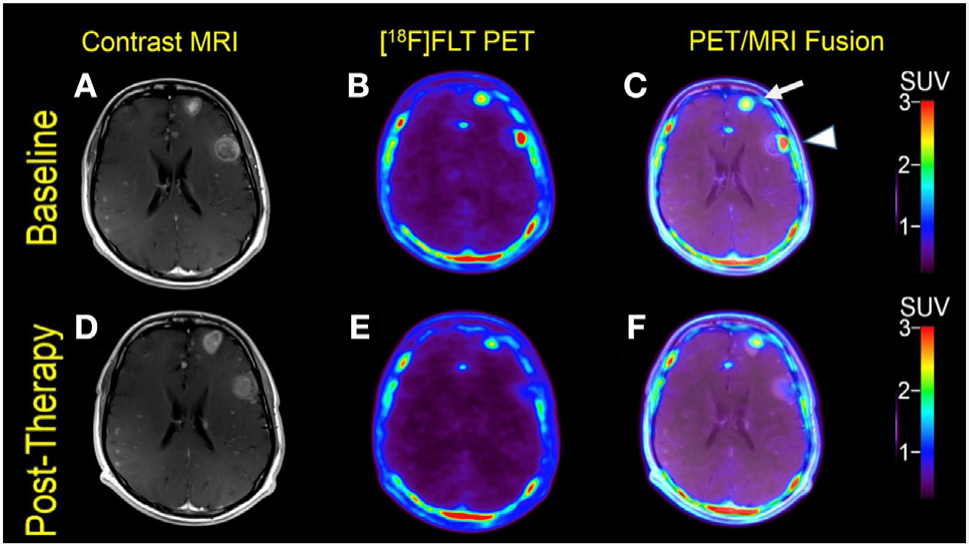
Case #2. Baseline magnetic resonance imaging (MRI) **(A)**, fluorothymidine (FLT)–positron-emission tomography (PET) **(B)**, and fused PET/MRI **(C)**; follow-up MRI **(D)**, FLT–PET **(E)**, and fused PET/MRI **(F)** at 1 month. Among multiple melanoma brain metastases, the left anterior frontal lobe lesion (arrow) showed a slight interval increase in FLT uptake and size [SUV ratio (SUVR) from 12.6 to 13.5; size from 1.7 to 2.0 cm]; the left lateral frontal lobe lesion (arrowhead) demonstrated interval decrease in FLT uptake from SUVR of 11.3–6.3, while the size remained stable at 2.3 cm. Same T1-weighted MRI parameters as in Figure 1.

**Figure 3:**
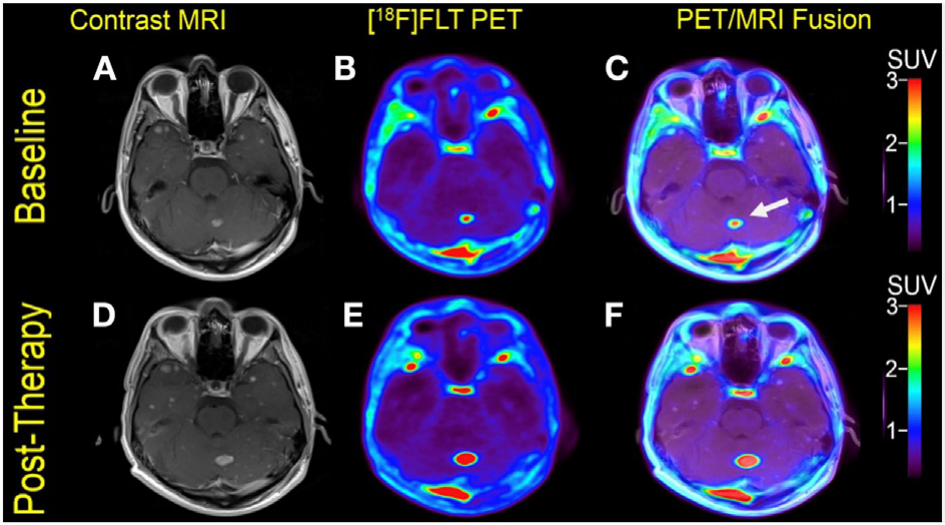
Case #2. Baseline magnetic resonance imaging (MRI) **(A)**, fluorothymidine (FLT)–positron-emission tomography (PET) **(B)**, and fused PET/MRI **(C)**; follow-up MRI **(D)**, FLT-PET **(E)**, and fused PET/MRI **(F)**. The left cerebellar lesion (arrow) showed interval increase in FLT uptake and size [SUV ratio from 11.2 to 18.9; size 1.0–1.5 cm].

Multiple larger cerebral metastases showed a variable mild to intense FLT uptake at baseline and showed a variable change in FLT proliferative activity, ranging from stable to marked reduction, at 1-month follow-up. Qualitative analysis suggested a mixed response as some lesions demonstrated interval decrease while others demonstrated interval increase in proliferative activity, but there was no evidence of new brain metastasis (Figures 2 and 3). The five measured lesions showed a median reduction of −44% (range −77 to +68%) in proliferative activity at 1-month follow-up (Table 2). The median SUVR was 11.3 (range 10.2–12.6) at baseline and 6.3 (range 2.4–18.9) at follow-up scan. On MRI, measured lesions showed +7% (range –64 to +50%) change in size. On a per-lesion basis, brain MR findings were overall concordant PET findings as lesions with interval size reduction became less metabolically active, and those with interval size increase became more metabolically active on PET. Also, many non-measurable lesions (<1 cm) grew larger but remained non-avid or showed only minimal FLT avidity on follow-up scan.

**Table 2:** Case #2, size (cm) and SUV ratio (SUVR) of 5 representative melanoma brain metastases.

Size (baseline)	Size (follow-up)	Change in %	SUVR (baseline)	SUVR (follow-up)	Change in %
1.4	0.5	−64	11.7	3.9	−67
1.7	2	18	12.6	13.5	7
2.3	2.3	0	11.3	6.3	−44
1	0.8	−20	10.2	2.4	−77
1	1.5	50	11.2	18.9	68

Standard-of-care brain MRI acquired near the time of the post-therapy research scan was consistent with the research MRI scan and was concerning for progression of brain metastases even though the patient was stable neurologically with occasional episodes of headache. A small liver metastasis was stable on clinical CT scans. Because of the brain MRI findings, however, the therapy was switched over to dabrafenib and trametinib, which led to a partial response of MBM. The patient is still alive 16 months following treatment initiation.

### Subjects with Baseline Scan Only

#### Case #3

**Figure 4:**
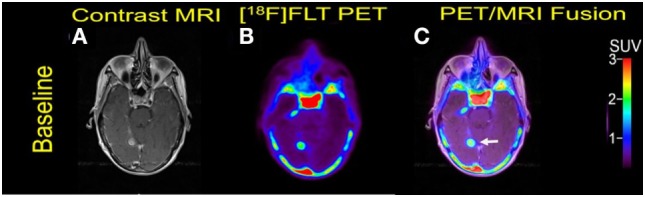
Case #3. Baseline magnetic resonance imaging (MRI) **(A)**, fluorothymidine (FLT)–positron-emission tomography (PET) **(B)**, and fused PET/MRI **(C)**. The 1.1 cm right posterior, medial temporal lobe lesion (arrow) showed FLT avidity with SUV ratio of 7.5.

A 70-year-old woman was initially diagnosed with nodular malignant melanoma in the left leg, 2.27 Breslow depth, stage T4N3M0, in March 2013. She developed multiple recurrences in the left calf and groin after complete lymph node dissection. Mutation analysis was positive for BRAF V600E. Brain metastasis was diagnosed 15 months after the initial diagnosis, and widespread bone metastasis was present at this time. She underwent Cyberknife radiosurgery to one of the two brain metastases and was initiated on combination therapy with Dabrafenib and trametinib. But she went to develop extracranial and intracranial disease progression and died 7 months after the initiation of targeted therapy. Two measurable MBM were noticed at baseline PET/MRI scan (Figure 4).

#### Case #4

**Figure 5:**
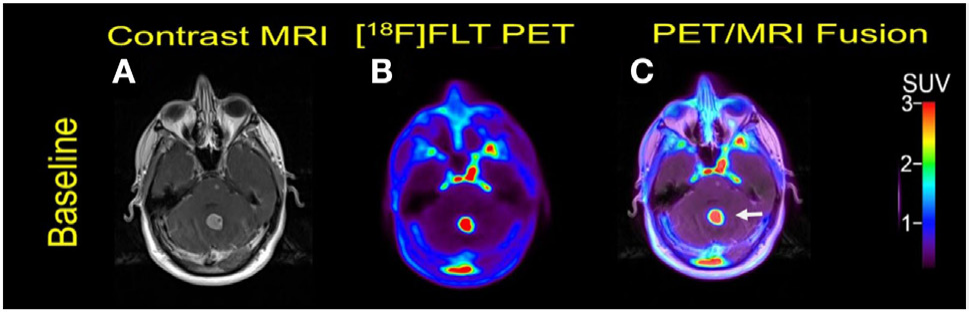
Case #4. Baseline magnetic resonance imaging (MRI) **(A)**, fluorothymidine (FLT)–positron-emission tomography (PET) **(B)**, and fused PET/MRI **(C)**. The 1.6-cm cerebellar lesion (arrow) with FLT avidity showed SUV ratio of 18.2.

A 27-year-old man was diagnosed with a scalp melanoma, Breslow thickness 1.2 cm, stage T1N0M0, in March 2010. Mutation analysis was positive for BRAF V600E. In November 2014, he presented with refractory headache and was diagnosed with brain metastases on MRI and pulmonary metastases on CT. He underwent surgical excision of two brain metastases and stereotactic radiosurgery using Gamma Knife^®^. Treatment with vemurafenib and cobimatinib was initiated in April 2015, which provided good extracranial and intracranial response but was ultimately discontinued for skin toxicity. The patient was initiated with standard-of-care pembrolizumab in August 2015 and was lost to follow-up after the October 2015 clinical visit. Three measurable MBM were present at baseline (Figure 5).

#### Case #5

**Figure 6:**
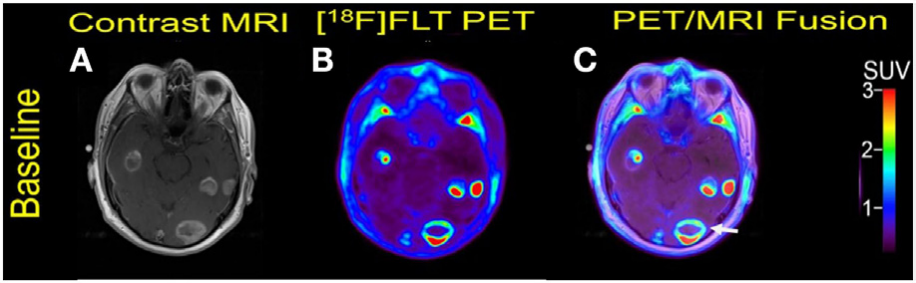
Case #5. Baseline magnetic resonance imaging (MRI) **(A)**, fluorothymidine (FLT)-positron-emission tomography (PET) **(B)**, and fused PET/MRI **(C)**. Multiple FLT avid brain metastases with Gadolinium contrast enhancement were present. The largest lesion in the left occipital lobe with central necrosis (arrow) measured 2.9 cm with SUV ratio of 14.9.

A 57-year-old male was initially diagnosed with abdominal malignant melanoma in 2007. A metastasis to the left breast was excised in 2009 followed by adjuvant temozolomide. In July 2011, he developed local recurrence in the left breast and was treated with interferon. Subsequent pulmonary metastases were surgically resected in 2013. In January 2014, he developed acute left upper extremity weakness, and a brain MRI showed extensive brain metastases. He underwent resection of a left frontal lobe metastasis which as positive for BRAF V600E mutation. A combination therapy with dabrafenib and trametinib was initiated in March 2014, but the patient died 5 months later due to disease progression. Multiple bilateral MBM were noticed at baseline (Figure 6).

The 11 contrast-enhancing MBM in these three subjects showed a median size 1.6 cm (range 1.0–2.9) and median SUVR 13.5 (range 7.5–18.4), Table 3. All measurable MBM were FLT avid in the current five subjects with baseline PET/MRI scan. The median size of the 22 measured MBM was 1.7 cm (range 1.0–2.9) and the SUVR was 9.9 (range 3.2–18.4).

**Table 3:** Size (cm) and SUV ratio (SUVR) of representative melanoma brain metastasis in three subjects that underwent baseline scan only.

Case #	Size (baseline)	SUVR (baseline)
3	1.1	7.5
	1.0	8.0
4	1.6	18.2
	1.0	15.6
	1.0	10.0
5	2.9	14.9
	2.1	15.4
	1.7	12.7
	1.9	12.2
	1.7	18.4
	1.0	13.5

## Discussion

Routine imaging for the treatment monitoring of brain metastases is usually based on contrast-enhanced T1-weighted MRI. However, the ability of conventional MRI to differentiate tumor tissue from post-therapeutic effects and pseudoprogression following therapies may be suboptimal. PET imaging can provide relevant additional information on the presence of residual viable tumor, which may allow for more accurate diagnosis, particularly in clinically equivocal situations. The current pilot study is the first FLT-PET study to explore the role of FLT–PET in patients with MBM undergoing targeted therapy and immunotherapy to date. In all five subjects, the MBM showed high proliferative activity at baseline with median SUVR of 9.9, which fulfills an important requirement for PET treatment monitoring (26). Although only two subjects returned for the follow-up scan that limits any inferences regarding the added value of FLT–PET to MRI, the proliferative and morphologic findings were concurrent following therapies, indicating that a combined PET/MRI exam may help improve diagnostic confidence and clinical management.

Case #1 received targeted systemic antitumor therapy with the combination of a BRAF inhibitor (dabrafenib) and a MEK inhibitor (trametinib). As a BRAF inhibitor, dabrafenib has demonstrated efficacy in active MBM. Disease control rate was 81% for patients without prior treatment and 89% for those with previous surgery, radiation or both, and the median overall survival was 33 and 31 weeks, respectively (27). In patients with metastatic melanoma but without MBM, the combination of dabrafenib and trametinib showed a median progression-free survival of 9.4 months, as compared to 5.8 months in the monotherapy group (*P* < 0.001). The rate of complete or partial response was 76% with combination therapy as compared to 54% with monotherapy (*P* = 0.03) (28). BRAF inhibitors combined with radiotherapy are being studied, with preliminary data showing potential improvement of 6-month survival to 92%, although this has not been confirmed in randomized studies (29).

In Case #1, all six measured lesions showed greater than 30% FLT reduction after 1 month of therapy indicating partial treatment response, modified from the PERCIST criteria (with the use of maximum SUV and SUVR instead of lesion peak SUV) (26). Although MRI findings point to the same direction of partial treatment response, only 3 of 6 lesions showed a size reduction greater than 30%, which is the cut-off for partial response based on RECIST 1.1 criteria (30). These morphologic findings are also consistent with the Macdonald criteria and RANO criteria developed specifically for treatment monitoring of brain lesions (31, 32). The remaining three lesions showed variable size reductions between −4 and −25%, which could be interpreted as stable disease morphologically. Similarly, the more consistent reduction in FLT proliferative activity compared with morphology has been demonstrated in patients with glioblastomas (19).

Case #2 received a combination of a PD1 inhibitor (nivolumab) and an anti-CTLA4 antibody (ipilimumab). Ipilimumab was the first checkpoint blockade immunotherapy shown to improve overall survival in metastatic melanoma patients. Nivolumab was also recently approved for advanced extracerebral melanoma due to its superiority to chemotherapy after disease progression on ipilimumab (33). Nivolumab and ipilimumab can be administered concurrently with a manageable safety profile.

In Case #2, the morphologic response was difficult to characterize because two of five measured lesions showed some size reduction (−20 and −64%), while two other lesions showed a size increase (18 and 50%), and the size of one lesion was unchanged (0%). These findings are consistent with the variable pattern of morphologic response demonstrated in previous reports (34–38). However, Case #2 shows largely concurrent morphologic and proliferative changes following immunotherapy with nivolumab and ipilimumab, with most lesions showing either interval increase or decrease in size and FLT uptake, which can be interpreted as mixed treatment response. The decision to switch the therapy over to dabrafenib and trametinib was based on radiographic findings, not because of neurological symptoms. An actual disease progression was not proven by biopsy at that time. Fortunately, the patient responded well after switching to dabrafenib and trametinib and is still alive to date.

The clinical benefit of FLT–PET has been demonstrated in glioma patients. Studies have shown that FLT–PET was able to separate real progression from radionecrosis (39, 40). In a study of 19 glioma patients treated with bevacizumab (humanized antibody against VEGF) and irinotecan (an inhibitor of topoisomerase I), Chen et al. (19) showed that responders, defined as those with a 25% or greater decrease in SUV, survived 3 times as long as non-responders (10.8 vs. 3.4 months); they concluded that FLT–PET was a better predictor of overall survival compared with MRI. In a retrospective FLT–PET study involving 21 patients with high-grade glioblastoma treated with surgery and chemoradiation, the average SUV ratio-to-normal brain of recurrent gliomas (7.01 ± 2.26) was statistically significantly higher than that of necrotic lesions (4.60 ± 1.23) (41). The findings of Case #2, albeit MBM instead of gliomas, are consistent with previous reports as the median SUVR before treatment was high at 9.8 (range 8.8–11.1) and dropped to a low level of 4.6 (range 1.1–8.5) at follow-up scan. Although the majority of literature reports on FLT–PET has been encouraging, some studies have also shown that FLT–PET may not be able to discriminate response to therapy from pseudoprogression in brain tumors (42, 43).

The desired response of effective cancer therapy is a reduction in tumor cell proliferation. At baseline scan, all measurable MBM in the current five cases were FLT avid indicating that the proliferative activity was high. An early measure of therapy efficacy as quantified by FLT–PET provides a molecular complement to the morphologic characterization afforded by MRI. A PET/MRI exam combining the evaluation of pathophysiology, metabolism, and morphology will improve the diagnostic confidence and accuracy for MBM. But more importantly, the added value of FLT–PET will be in the evaluation of treatment response as morphologic MRI has limitations in distinguishing pseudoprogression from true progression (12, 35). In melanoma patients treated with immunotherapy, the radiographic pseudoprogression is highest among cancers and may be seen in 10–15% of patients (12), compared to 2–3% in lung cancer and squamous cell carcinoma of the head and neck (44, 45). Immunotherapy for melanoma is promising, but because the mechanisms of therapeutic immunologic response are different than systemic cytotoxic chemotherapy, the use of advanced imaging techniques such as hybrid PET/MRI can be used to provide a better understanding of the molecular and anatomical features confounding accurate response assessment. Potentially, MBM may show a proliferative reduction on PET despite apparent morphologic progression (i.e., pseudoprogression) on MRI. In this context, PET/MRI may shed light on the unique clinical responses associated with immunotherapy, which may have a positive impact on clinical decisions during the course of therapy and serve as an effective tool to assess for long-term disease outcome.

Moreover, advanced MRI techniques, such as diffusion-weighted imaging, MR spectroscopy, and perfusion MRI, may provide further improvement in biochemical characterization of tissues (46). Thus, future studies applying multi-parametric PET/MRI biomarkers could lead to effective personalized strategies for treatment monitoring. The concern about the medical cost associated with advanced PET imaging has been raised. O-(2-18F-fluoroethyl)-l-tyrosine PET, however, has been shown to be cost-effective for the treatment monitoring of glioma patients undergoing antiangiogenic therapy, which helps avoid costs related to overtreatments as well as decrease patient side effects (47).

We acknowledge the limitations of our case series in which only two of 5 (40%) enrolled patients returned for the follow-up scan. This low retention rate might not be surprising considering the advanced disease with dismal clinical outcome in these subjects. Retention rates appear more favorable in less aggressive malignancies such as prostate cancer, which may range from 74.6 to 86.0% (48). In the analysis of the PET data (due to a system malfunction that prevented us from retrieving sinogram or list mode PET data) instead of performing a kinetic analysis or a late-time-frame analysis, we were forced to employ the non-standard strategy described. We have attempted to compensate for using data averaged over the full acquisition time, which includes tracer in the vascular phase, by normalizing to normal brain tissue (pons), which is dominated by the vascular phase.

The five cases presented here indicate that hybrid FLT–PET/MRI may be useful to diagnose MBM and to monitor treatment response to targeted therapy and immunotherapy in patients with MBM. However, further studies are required to elucidate the significance of these findings and to unravel the diagnostic potential of hybrid PET/MRI for MBM therapy evaluation and in distinguishing true progression from pseudoprogression.

## Ethics Statement

This study was carried out in accordance with the recommendations of the University of Pittsburgh Institutional Review Board, with written informed consent from all subjects. All subjects provided a written informed consent for their participation in this study and for their personal information to be used for research and publication. Written informed consent was obtained in accordance with the Declaration of Helsinki.

## Author Contributions

NN: data analysis and interpretation, manuscript writing, accountable for all aspects of the work, and manuscript approval. MY: patient enrollment, manuscript revision, integrity of the work, and manuscript approval. AT: data analysis, manuscript revision, integrity of the work, and manuscript approval. JK: patient enrollment, manuscript revision, integrity of the work, and manuscript approval. HT: patient enrollment, study design, manuscript revision, integrity of the work, and manuscript approval. JM: study design, patient enrollment, data interpretation, manuscript writing, accountable for all aspects of the work, and manuscript approval.

## Conflict of Interest Statement

The authors declare that the research was conducted in the absence of any commercial or financial relationships that could be construed as a potential conflict of interest.
